# Distinct trajectories of disease-specific health status in heart failure patients undergoing cardiac resynchronization therapy

**DOI:** 10.1007/s11136-015-1176-3

**Published:** 2015-11-13

**Authors:** Mirjam H. Mastenbroek, Susanne S. Pedersen, Mathias Meine, Henneke Versteeg

**Affiliations:** Department of Cardiology, University Medical Center, Heidelberglaan 100, 3508 GA Utrecht, The Netherlands; CoRPS - Center of Research on Psychology in Somatic Diseases, Tilburg University, Tilburg, The Netherlands; Department of Psychology, University of Southern Denmark, Odense, Denmark; Department of Cardiology, Odense University Hospital, Odense, Denmark; Department of Cardiology, Thoraxcenter, Erasmus Medical Center, Rotterdam, The Netherlands

**Keywords:** Heart failure, Cardiac resynchronization therapy, Health status, KCCQ, Trajectories

## Abstract

**Purpose:**

It is well known that a significant proportion of heart failure patients (10–44 %) do not show improvement in symptoms or functioning from cardiac resynchronization therapy (CRT), yet no study has examined patient-reported health status trajectories after implantation.

**Methods:**

A cohort of 139 patients with a CRT-defibrillator (70 % men; age 65.7 ± 10.1 years) completed the Kansas City Cardiomyopathy Questionnaire (KCCQ) prior to implantation (baseline) and at 2, 6, and 12–14 months post-implantation. Latent class analyses were used to identify trajectories and associates of disease-specific health status over time.

**Results:**

All health status trajectories showed an initial small to large improvement from baseline to 2-month follow-up, whereafter most trajectories displayed a stable pattern between short- and long-term follow-up. Low educational level, NYHA class III/IV, smoking, no use of beta-blockers, use of psychotropic medication, anxiety, depression, and type D personality were found to be associated with poorer health status in unadjusted analyses. Interestingly, subgroups of patients (12–20 %) who experienced poor health status at baseline improved to stable good health status levels after implantation.

**Conclusions:**

Levels of disease-specific health status vary considerably across subgroups of CRT-D patients. Classification into poorer disease-specific health status trajectories was particularly associated with patients’ psychological profile and NYHA classification. The timely identification of CRT-D patients who present with poor disease-specific health status (i.e., KCCQ score < 50) and a distressed psychological profile (i.e., anxiety, depression, and/or type D personality) is paramount, as they may benefit from cardiac rehabilitation in combination with psychological intervention.

## Introduction

Heart failure (HF) is a complex and debilitating clinical syndrome, characterized by symptoms of fatigue, dyspnea, diminished exercise capacity, fluid retention, reduced quality of life, and reduced survival [1]. Cardiac resynchronization therapy (CRT), with or without an implantable cardioverter-defibrillator, is a well-established treatment in selected patients with drug-refractory HF and an electrical conduction delay [2]. Several large-scale randomized controlled trials have demonstrated that CRT improves not only prognosis, but also patient-reported health status [3, 4].

Patient-reported health status, including symptoms, functioning, and health-related quality of life, has become an increasingly important outcome measure in cardiac patients [5]. Thus far, the majority of studies on health status in CRT and HF patients reported on prevalence rates or change in mean scores over time [3]. However, change in health status of the total sample is fairly meaningless if there are in fact multiple subgroups that have different means and different patterns of change over time (i.e., trajectories) [6]. Although it is well known that a significant proportion of patients (10–44 %) do not show improvement in symptoms or functioning from CRT [7], no study to date has examined patient-reported health status trajectories after implantation.

Latent class analysis would permit the identification of patients reporting persistently low health status, who need additional care above and beyond standard HF management. This is of utmost importance, since poor patient-reported health status has been shown to predict mortality and rehospitalization in HF patients independent of traditional risk factors [8]. Additionally, CRT studies have shown that the majority of established outcome measures, including New York Heart Association (NYHA) functional class and echocardiographic and hemodynamic parameters, are only marginally associated with patient-reported outcomes [9]. By contrast, the role of psychological factors has largely been neglected in this context, although such factors may contribute more to differences in patient-reported outcomes [9]. Knowing which factors are characteristics of patients with (persistently) reduced patient-reported health status after implantation may provide targets for intervention, thereby improving the clinical response to CRT.

Hence, the goal of the current study was to identify the trajectories and demographic, clinical, and psychological associates of disease-specific health status in the first 14 months after CRT-defibrillator (CRT-D) implantation.

## Methods

### Study design and participants

The sample comprised HF patients receiving a first-time CRT-D between January 2009 and August 2011 at the University Medical Center Utrecht (UMCU), the Netherlands. All patients participated in the ‘The influence of PSYchological factors on health outcomes in HEART failure patients treated with Cardiac Resynchronization Therapy (PSYHEART-CRT): A prospective, single-center, observational study’. Patients were not eligible for participation: when aged <18 or >85 years; if they had insufficient knowledge of the Dutch language; a history of psychiatric illness other than affective/anxiety disorders; cognitive impairments; or if they were on the waiting list for heart transplantation. Patients were asked to complete a set of standardized and validated questionnaires 1 day prior to implantation (baseline) and 2, 6, and 12–14 months after implantation. The study protocol was approved by the Medical Ethics Committee of the UMCU. The study was conducted in accordance with the Helsinki Declaration.

### Measures

#### Demographic and clinical variables

Information on demographic and clinical characteristics was captured via purpose-designed questions in the baseline questionnaire and/or via patients’ medical records.

#### Patient-reported health status

The Kansas City Cardiomyopathy Questionnaire (KCCQ) was used to assess HF-specific health status [10]. The KCCQ is a 23-item self-report questionnaire that taps into the following dimensions: physical limitation, symptoms, social function, and quality of life. In the current study, the following summary scores within the KCCQ were calculated: clinical summary score (KCCQ-CS), quality of life (KCCQ-QoL), and social limitation (KCCQ-SL). The KCCQ-CS is the mean of the physical limitation and symptoms score, thus representing physical health status. Scores are transformed into a score from 0 to 100, with higher scores representing less physical or social limitation, less symptoms, or better quality of life. Poor health status is defined as a KCCQ (sub)scale score of <50 points. The minimal clinically meaningful difference in KCCQ scores is 5 points [11]. The validity and reliability of the KCCQ have previously been established and the measure was shown to be highly sensitive to clinical change in HF patients [10, 11].

#### Psychological variables and personality

The Patient Health Questionnaire (PHQ-9) was used to measure depressive symptoms at baseline. This is a nine-item questionnaire with the items mirroring the diagnostic criteria for major depressive disorder. Patients are asked to rate how often each symptom has bothered them during the past 2 weeks on a scale from 0 (‘*not at all*’) to 3 (‘*nearly every day*’) (score range 0–27). Patients who score ≥10 points are considered to have moderate or severe depressive symptoms [12]. The PHQ-9 is brief, responsive to change over time, and has good reliability and validity in medical outpatients and patients with HF.

 The state anxiety subscale of the State-Trait Anxiety Inventory (STAI-S) was used to measure baseline symptoms of anxiety [13]. All items are rated on a four-point Likert scale ranging from 1 (‘*not at all*’) to 4 (‘*very much so*’) (score range 20–80). Higher scores indicate higher levels of anxiety. A cutoff score ≥ 40 indicates probable clinical levels of anxiety. The STAI-S has shown to be a valid and reliable measure [13].

The 14-item type D scale (DS14) was administered at baseline to assess Type D personality, which is defined as the tendency to experience negative emotions across time and situations paired with the tendency to inhibit these emotions [14]. The DS14 comprises two subscales, ‘negative affectivity’ (e.g., ‘*I often feel unhappy*’) and ‘social inhibition’ (e.g., ‘*I am a closed kind of person*’), each consisting of seven items. Items are answered on a five-point Likert scale ranging from 0 (‘*false*’) to 4 (‘*true*’), with total scores ranging from 0 to 28 for both subscales. A standardized cutoff score of ≥ 10 on both subscales was used to identify patients with a Type D personality [15]. The DS14 is a valid and reliable scale [14].

### Statistical analyses

All patients (*n* = 139) had at least 1 measurement of health status and were included in the analyses. With respect to physical health status, 112 (81 %), 13 (9 %), 12 (9 %), and 2 (1 %) patients had 4, 3, 2, and 1 measurement(s), respectively. With respect to quality of life, 111 (80 %), 13 (9 %), 13 (9 %), and 2 (1 %) had 4, 3, 2, and 1 measurement(s), respectively. With respect to social limitation, 96 (69 %). 25 (18 %), 15 (11 %), and 3 (2 %) had 4, 3, 2, and 1 measurement(s), respectively. All available data were used in the analyses.

 Latent GOLD 5.0 [16] was used to fit a number of latent class regression models in order to determine how many latent classes (i.e., health status trajectories) could be identified. Time was entered as a nominal predictor, whereas health status (KCCQ-CS, KCCQ-QoL, or KCCQ-SL) was treated as continuous outcome. For each dependent variable (i.e., KCCQ-CS, KCCQ-QoL, and KCCQ-SL), eight models were compared with an increasing number of trajectories (1–8 trajectories). To determine the optimal number of trajectories, the Bayesian information criterion (BIC) was used. The BIC is a criterion for model selection among a finite set of models, with a lower BIC indicating a better fit. In case of a difference in BIC of <3 between two consecutive models, the least complex model was preferred (i.e., with the lowest number of trajectories). Subsequently, SPSS 20.0 for Windows (SPSS Inc., Chicago, IL, USA) was used to determine which variables were univariately associated with health status class membership, while the corresponding *p* values were obtained using the Step-3-Dependent analysis procedure in Latent GOLD which corrects for classification error to prevent bias [16]. This correction is performed by obtaining estimates of the number of classification errors when assigning individuals to latent classes, which enables for proportional assignment in which individuals are treated as belonging to each of the classes with weights equal to the posterior membership probabilities. Step-3-Dependent analysis yields a separate bivariate analysis for each dependent variable (i.e., demographic, clinical, or psychological variable), which is similar to cross-tabulations (for categorical variables) and ANOVAs (for continuous variables). The Wald (=) statistic, which tests the equality of each set of regression effects across classes, was used to evaluate statistical significance. In order to correct for multiple comparisons, the Bonferroni correction was applied.

## Results

### Patient characteristics

The sample comprised 139 CRT-D patients with a mean age of 65.7 ± 10.1 years, and 97 (70 %) patients were male. The underlying HF etiology was ischemic in 49 % of the patients, and 21, 77, and 2 % of patients were classified as having NYHA functional class II, III, and IV, respectively. Forty-three percent of patients reported clinically relevant levels of anxiety, 23 % of patients experienced moderate to severe symptoms of depression, and 23 % of patients were classified as having a Type D personality. Complete information on the demographic and clinical characteristics of the patient sample is given in Table 1.

**Table 1:** Identification of the number of latent classes for health status after CRT-D implantation

Solution	Physical health status			Quality of life			Social limitation		
	LL	Npar	BIC	LL	Npar	BIC	LL	Npar	BIC
1 Class	−2334.13	5	4692.93	−2367.98	5	4760.63	−2359.51	5	4743.70
2 Classes	−2212.71	11	4479.69	−2286.94	11	4628.16	−2255.51	11	4565.29
3 Classes	−2176.28	17	4436.44	−2259.92	17	4603.73	−2217.67	17	4519.24
4 Classes	−2152.69	23	4418.87	−2239.63	23	4592.75	−**2191.37**	**23**	**4496.22**
*5 Classes*	−**2135.60**	**29**	**4414.30**	−**2221.30**	**29**	**4585.70**	−2177.38	*29*	4497.87
6 Classes	−2124.26	35	4421.23	−2210.51	35	4593.72	−2166.43	35	4505.57
7 Classes	−2112.40	41	4427.12	−2197.05	41	4596.41	−2153.72	41	4509.76
8 Classes	−2103.08	47	4438.08	−2184.38	47	4600.68	−2145.69	47	4523.31

*LL* log likelihood; *Npar* number of parameters; *BIC* Bayesian information criterion

The selected number of classes is marked in bold for each outcome

### Trajectories of physical health status (KCCQ-CS)

Based on the BIC, a five-class model was found to be the best fitting model for physical health status (Table 1). The class sizes of the physical health status trajectories varied between 14 (10 %) and 41 (29 %) patients, and the trajectories explained 71 % of the total variance in KCCQ-CS scores. Trajectories differed from each other with respect to intercept (Wald (=) 355.47, *p* < .001) and time slope (Wald (=) 200.69, *p* < .001).

**Fig. 1:**
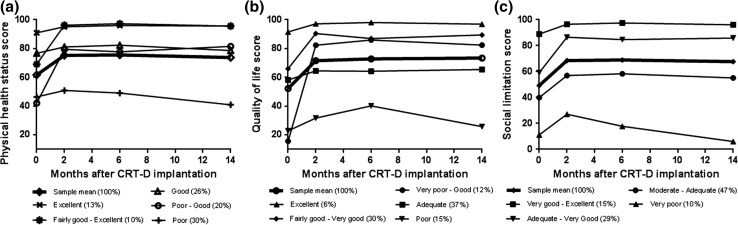
**a**–**c** Trajectories of physical health status, quality of life, and social limitation after CRT-D implantation

All five KCCQ-CS trajectories showed an initial improvement from baseline to 2-month follow-up (Fig. 1a). Three of them showed a small increase, whereas two trajectories showed a large increase (37.3 and 27.1 points on the KCCQ-CS for the ‘Poor-Good’ and ‘Fairly good-Excellent’ trajectory, respectively). After this initial improvement in physical health status, patients in all but one trajectory showed subsequent stability between short- and long-term follow-up. Four trajectories showed stable good to excellent physical health status, whereas patients in the ‘Poor’ trajectory (comprising the largest group of patients (29 %)) reported poor physical health status that deteriorated even more during long-term follow-up, with a mean KCCQ-CS score between 50.9 and 40.9 over time.

Demographic, clinical, and psychological characteristics at baseline stratified by KCCQ-CS status class are given in Table 2. Low educational level, NYHA class III/IV, and depression were associated with poorer physical health status. The relationship between ICD shock and physical health status was less clear. The highest percentages of ICD shock were found in patients in the ‘Poor’ (13 %) and ‘Good’ trajectories (17 %).

**Table 2:** Baseline characteristics stratified by physical health status trajectories after CRT-D implantation*

Baseline variable	Missing	Total	Excellent	Fairly good–Excellent	Good	Poor–Good	Poor	*p* value^†^
	(*n*, %)	(*n* = 139)	(*n* = 18)	(*n* = 14)	(*n* = 38)	(*n* = 28)	(*n* = 41)	
*Demographics*								
Mean age (SD)	0 (0)	65.7 (10.1)	68.0 (9.9)	63.4 (12.7)	64.8 (11.3)	64.8 (7.9)	66.8 (9.6)	.64
Male gender	0 (0)	97 (70)	16 (89)	10 (71)	30 (79)	13 (46)	28 (68)	.019
Having a partner	0 (0)	113 (81)	17 (94)	12 (86)	32 (84)	22 (79)	30 (73)	.45
Low education^‡^	1 (1)	18 (13)	0 (0)	1 (7)	1 (3)	7 (25)	9 (23)	**<.001**
Being employed	1 (1)	30 (22)	5 (28)	4 (29)	11 (29)	2 (7)	8 (20)	.51
*Clinical variables*								
Upgrade^§^	0 (0)	36 (26)	5 (28)	4 (29)	9 (24)	7 (25)	11 (27)	.98
ICD as sec. prevention	0 (0)	26 (19)	7 (39)	2 (15)	5 (13)	6 (21)	6 (15)	.34
Ischemic etiology	0 (0)	68 (49)	10 (56)	3 (21)	20 (53)	13 (46)	22 (54)	.29
NYHA class III/IV	0 (0)	110 (79)	6 (33)	10 (71)	28 (74)	28 (100)	38 (93)	**<.001**
Mean LVEF (SD)	6 (4)	24.9 (8.6)	27.1 (10.2)	23.2 (7.7)	24.2 (8.7)	26.2 (8.6)	24.4 (8.2)	.74
Mean QRS (SD)	11 (8)	162 (25)	163 (24)	166 (22)	162 (28)	165 (26)	160 (24)	.92
LBBB	28 (20)	60 (54)	8 (50)	8 (73)	17 (50)	14 (61)	13 (48)	.51
ESV responder^||^	25 (18)	61 (54)	10 (63)	7 (58)	23 (66)	10 (46)	11 (38)	.40
Comorbidity^#^	6 (4)	79 (59)	8 (47)	4 (31)	24 (63)	17 (63)	26 (68)	.55
ICD shock	18 (13)	11 (9)	0 (0)	1 (8)	6 (17)	0 (0)	4 (13)	**<.001**
Smoking	0 (0)	21 (15)	1 (6)	1 (7)	5 (13)	7 (25)	7 (17)	.43
*Medication use*								
Amiodarone	0 (0)	17 (12)	4 (22)	2 (14)	3 (8)	1 (4)	7 (17)	.38
ACE inhibitors	0 (0)	126 (91)	16 (89)	11 (79)	36 (95)	25 (89)	38 (93)	.58
Beta-blockers	0 (0)	108 (78)	15 (83)	10 (71)	31 (82)	23 (82)	29 (71)	.60
Statins	0 (0)	84 (60)	14 (78)	3 (21)	20 (53)	19 (68)	28 (68)	.018
Psychotropic medication	0 (0)	33 (24)	1 (6)	1 (7)	6 (16)	9 (32)	16 (39)	.041
*Psychological functioning*								
Anxiety (STAI-S ≥ 40)	2 (1)	59 (43)	2 (11)	3 (21)	12 (32)	17 (61)	25 (63)	.001
Depression (PHQ-9 ≥ 10)	1 (1)	32 (23)	0 (0)	2 (14)	1 (3)	8 (29)	21 (51)	**<.001**
Type D personality	1 (1)	32 (23)	1 (6)	1 (8)	8 (21)	8 (29)	14 (34)	.13

*ACE* angiotensin-converting enzyme; *ESV* end-systolic volume; *ICD* implantable cardioverter-defibrillator; *LBBB* left bundle branch block; *NYHA* New York Heart Association; *SD* standard deviation; *sec.* secondary

* Results are presented as *n* (%), unless otherwise indicated. Significant results are printed in bold (Bonferroni’s corrected *p* value = 0.05/72 = 6.9e^−4^)

^†^ *p* values were obtained using adjusted Step-3-*Dependent* analysis procedures in Latent GOLD 5.0. All other values were observed values obtained using SPSS

^‡^Defined as primary education versus secondary education and higher

^§^Upgrade from another implantable device, either (biventricular) pacemaker or ICD without cardiac resynchronization therapy

^||^Echocardiographic response was defined as a decrease in left ventricular end-systolic volume of ≥15 % measured at 6-month follow-up

^#^Comorbidity = atrial fibrillation, chronic obstructive pulmonary disease, diabetes, or renal failure

As shown in Fig. 1a, two classes of patients had a mean KCCQ-CS score <50 prior to implantation, which is defined as poor physical health status. At follow-up, patients in the ‘Poor-Good’ trajectory reported a strong improvement in physical health status in the first 2 months, whereas patients in the ‘Poor’ trajectory reported further deterioration. Post hoc analyses revealed a trend for gender, with proportionally more men in the poor physical health status trajectory as in the improving physical health status trajectory (68 vs. 46 %, *p* = .069). Furthermore, a trend was found for depression, with patients in the poor physical health status trajectory having a higher prevalence of depression prior to implantation as compared with patients in the improving physical health status trajectory (51 vs. 29 %, *p* = .061).

### Trajectories of quality of life (KCCQ-QoL)

With reference to quality of life, also a five-class model was preferred (Table 1). The class sizes of the quality of life trajectories varied between 9 (6 %) and 50 (36 %), and the trajectories explained 74 % of the total variance in KCCQ-QoL scores. Trajectories differed from each other with respect to intercept (Wald (=) 416.06, *p* < .001) and time slope (Wald (=) 218.26, *p* < .001).

All KCCQ-QoL trajectories showed an initial improvement from baseline to 2-month follow-up (Fig. 1b). Three of them showed a moderate increase (between 5.7 and 8.8 points on the KCCQ-QoL), whereas two trajectories showed a large increase (66.7 and 24.4 points on the KCCQ-QoL for the ‘Very poor-Good’ and ‘Fairly good-Very good’ trajectory, respectively). After this initial improvement in quality of life, all but one trajectory showed subsequent stability between short- and long-term follow-up, whereas patients in the ‘Poor’ trajectory reported poorer quality of life at 14-month follow-up compared with 6-month follow-up. The ‘Adequate’ trajectory comprised the largest group of patients (37 %), with a mean score between 58.2 and 65.5 over time.

Demographic, clinical, and psychological characteristics at baseline stratified by KCCQ-QoL status class are given in Table 3. Low educational level, smoking, no use of beta-blockers, use of psychotropic medication, anxiety, depression, and Type D personality were associated with poorer quality of life. The relationship between use of amiodarone and quality of life was less clear. The highest percentages of amiodarone use were found in the ‘Poor’ (30 %) and ‘Excellent’ trajectories (22 %), whereas no patients in the ‘Very poor-Good’ trajectory used amiodarone.

**Table 3:** Baseline characteristics stratified by quality of life trajectories after CRT-D implantation*

Baseline variable	Missing	Total	Excellent	Fairly good–Very good	Adequate	Very poor–Good	Poor	*p* value^†^
	(*n*, %)	(*n* = 139)	(*n* = 9)	(*n* = 42)	(*n* = 50)	(*n* = 18)	(*n* = 20)	
*Demographics*								
Mean age (SD)	0 (0)	65.7 (10.1)	68.1 (9.9)	67.1 (10.0)	63.8 (10.8)	64.7 (8.8)	67.1 (9.9)	.79
Male gender	0 (0)	97 (70)	7 (78)	31 (74)	36 (72)	10 (56)	13 (65)	.50
Having a partner	0 (0)	113 (81)	8 (89)	36 (86)	39 (78)	16 (89)	14 (70)	.57
Low education^‡^	1 (1)	18 (13)	0 (0)	2 (5)	5 (10)	6 (33)	5 (26)	**<.001**
Being employed	1 (1)	30 (22)	2 (22)	7 (17)	16 (33)	3 (17)	2 (10)	.24
*Clinical variables*								
Upgrade^§^	0 (0)	36 (26)	2 (22)	11 (26)	15 (30)	1 (6)	7 (35)	.13
ICD as sec. prevention	0 (0)	26 (19)	3 (33)	8 (19)	8 (16)	2 (11)	5 (25)	.44
Ischemic etiology	0 (0)	68 (49)	6 (67)	18 (43)	26 (52)	9 (50)	9 (45)	.76
NYHA class III/IV	0 (0)	110 (79)	2 (22)	28 (67)	45 (90)	17 (94)	18 (90)	.001
Mean LVEF (SD)	6 (4)	24.9 (8.6)	28.2 (11.3)	24.7 (8.5)	24.4 (9.0)	25.1 (7.9)	25.2 (7.5)	.98
Mean QRS (SD)	11 (8)	162 (25)	156 (20)	168 (26)	160 (26)	161 (22)	162 (26)	.39
LBBB	28 (20)	60 (54)	5 (56)	21 (58)	18 (45)	10 (71)	6 (50)	.53
ESV responder^||^	25 (18)	61 (54)	6 (67)	20 (54)	21 (50)	10 (71)	4 (33)	.44
Comorbidity^#^	6 (4)	79 (59)	4 (44)	24 (60)	28 (58)	10 (59)	13 (68)	.92
ICD shock	18 (13)	11 (9)	0 (0)	2 (5)	7 (16)	1 (7)	1 (7)	.009
Smoking	0 (0)	21 (15)	0 (0)	5 (12)	7 (14)	6 (33)	3 (15)	**<.001**
*Medication use*								
Amiodarone	0 (0)	17 (12)	2 (22)	5 (12)	4 (8)	0 (0)	6 (30)	**<.001**
ACE inhibitors	0 (0)	126 (91)	9 (100)	36 (86)	47 (94)	16 (89)	18 (90)	.021
Beta-blockers	0 (0)	108 (78)	9 (100)	33 (79)	42 (84)	12 (67)	12 (60)	**<.001**
Statins	0 (0)	84 (60)	6 (67)	24 (57)	32 (64)	10 (56)	12 (60)	.97
Psychotropic medication	0 (0)	33 (24)	0 (0)	6 (14)	16 (32)	3 (17)	8 (40)	**<.001**
*Psychological functioning*								
Anxiety (STAI-S ≥ 40)	2 (1)	59 (43)	1 (11)	10 (24)	22 (45)	12 (71)	14 (70)	**<.001**
Depression (PHQ-9 ≥ 10)	1 (1)	32 (23)	0 (0)	0 (0	13 (26)	8 (44)	11 (55)	**<.001**
Type D personality	1 (1)	32 (23)	0 (0)	6 (14)	10 (20)	7 (39)	9 (45)	**<.001**

*ACE* angiotensin-converting enzyme; *ESV* end-systolic volume; *ICD* implantable cardioverter-defibrillator; *LBBB* left bundle branch block; *NYHA* New York Heart Association; *SD* standard deviation; *sec.* secondary

* Results are presented as *n* (%), unless otherwise indicated. Significant results are printed in bold (Bonferroni’s corrected *p* value = 0.05/72 = 6.9e^−4^)

^†^ *p* values were obtained using adjusted Step-3-*Dependent* analysis procedures in Latent GOLD 5.0. All other values were observed values obtained using SPSS

^‡^Defined as primary education versus secondary education and higher

^§^Upgrade from another implantable device, either (biventricular) pacemaker or ICD without cardiac resynchronization therapy

^||^Echocardiographic response was defined as a decrease in left ventricular end-systolic volume of ≥15 % measured at 6-month follow-up

^#^Comorbidity = atrial fibrillation, chronic obstructive pulmonary disease, diabetes, or renal failure

As shown in Fig. 1b, two classes of patients had a mean KCCQ-QoL score around 20 points prior to implantation, which represents very poor quality of life. At follow-up, patients in the ‘Very poor-Good’ trajectory reported an enormous improvement in quality of life over time, whereas patients in the ‘Poor’ trajectory reported initial improvement, but lost their gain in quality of life between 6- and 14-month follow-up. Post hoc analyses showed that patients in the 'Poor' trajectory more often used amiodarone prior to implantation than patients in the 'Very poor-Good' trajectory (30 vs. 0 %, *p* = .021). Furthermore, a trend was found for echocardiographic response, with patients in the improving quality of life trajectory more often showing echocardiographic response after 6 months of CRT as compared with patients in the deteriorating quality of life trajectory (71 vs. 33 %, *p* = .052).

### Trajectories of social limitation (KCCQ-SL)

Based on the BIC, a four-class model was preferred for social limitation (Table 1). The class sizes of the social limitation trajectories varied between 11 (8 %) and 66 (47 %), and the trajectories explained 66 % of the total variance in KCCQ-SL scores. Trajectories differed from each other with respect to intercept (Wald (=) 462.73, *p* < .001) and time slope (Wald (=) 34.90, *p* < .001).

All KCCQ-SL trajectories showed an initial improvement from baseline to 2-month follow-up, ranging from 7.6 to 27.3 points (Fig. 1c). After this initial improvement in social limitation, all but one trajectory showed subsequent stability between short- and long-term follow-up, whereas patients in the ‘Very poor’ trajectory reported increased social limitations during follow-up. The ‘Moderate-Adequate’ trajectory comprised the largest group of patients (47 %), with a mean score between 39.9 and 58.3 over time.

Demographic, clinical, and psychological characteristics at baseline stratified by KCCQ-SL status class are given in Table 4. The KCCQ-SL classes showed different percentages according to NYHA class III/IV, ICD shock, and psychotropic medication. NYHA class III/IV and use of psychotropic medication were associated with more social limitation. The relationship between ICD shock and social limitation was less clear. The highest percentages of ICD shock were found in patients in the ‘Moderate-Adequate’ trajectory (17 %), whereas in all other trajectories none or only 5 % of patients received a shock.

**Table 4:** Baseline characteristics stratified by social limitation trajectories after CRT-D implantation*

Baseline variable	Missing	Total	Very good–Excellent	Adequate–Very good	Moderate–Adequate	Very poor	*p* value^†^
	(*n*, %)	(*n* = 139)	(*n* = 21)	(*n* = 41)	(*n* = 66)	(*n* = 11)	
*Demographics*							
Mean age (SD)	0 (0)	65.7 (10.1)	64.1 (10.7)	66.9 (8.8)	65.3 (10.8)	66.4 (10.5)	.89
Male gender	0 (0)	97 (70)	15 (71)	26 (63)	48 (73)	8 (73)	.64
Having a partner	0 (0)	113 (81)	19 (91)	34 (83)	53 (80)	7 (64)	.46
Low education^‡^	1 (1)	18 (13)	2 (10)	2 (5)	12 (19)	2 (18)	.30
Being employed	1 (1)	30 (22)	6 (29)	9 (23)	13 (20)	2 (18)	.74
*Clinical variables*							
Upgrade^§^	0 (0)	36 (26)	4 (19)	13 (32)	16 (24)	3 (27)	.79
ICD as sec. prevention	0 (0)	26 (19)	7 (33)	6 (15)	9 (14)	4 (36)	.05
Ischemic etiology	0 (0)	68 (49)	13 (62)	14 (34)	36 (55)	5 (46)	.15
NYHA class III/IV	0 (0)	110 (79)	8 (38)	32 (78)	61 (92)	9 (82)	**<.001**
Mean LVEF (SD)	6 (4)	24.9 (8.6)	28.3 (9.7)	23.9 (7.9)	24.5 (8.6)	24.8 (8.7)	.38
Mean QRS (SD)	11 (8)	162 (25)	162 (27)	168 (26)	160 (23)	158 (27)	.82
LBBB	28 (20)	60 (54)	12 (63)	21 (58)	24 (49)	3 (43)	.53
ESV responder^||^	25 (18)	61 (54)	12 (63)	21 (57)	27 (53)	1 (14)	.40
Comorbidity^#^	6 (4)	79 (59)	8 (38)	23 (59)	43 (69)	5 (46)	.08
ICD shock	18 (13)	11 (9)	0 (0)	2 (5)	9 (17)	0 (0)	**<.001**
Smoking	0 (0)	21 (15)	1 (5)	6 (15)	12 (18)	2 (18)	.50
*Medication use*							
Amiodarone	0 (0)	17 (12)	3 (14)	3 (7)	6 (9)	5 (46)	.032
ACE inhibitors	0 (0)	126 (91)	17 (81)	38 (93)	60 (91)	11 (100)	.40
Beta-blockers	0 (0)	108 (78)	18 (86)	32 (78)	53 (80)	5 (46)	.046
Statins	0 (0)	84 (60)	14 (67)	24 (59)	39 (59)	7 (64)	.94
Psychotropic medication	0 (0)	33 (24)	0 (0)	5 (12)	24 (36)	4 (36)	**<.001**
*Psychological functioning*							
Anxiety (STAI-S ≥ 40)	2 (1)	59 (43)	4 (19)	16 (36)	31 (48)	8 (73)	.031
Depression (PHQ-9 ≥ 10)	1 (1)	32 (23)	1 (5)	3 (7)	22 (33)	6 (55)	.002
Type D personality	1 (1)	32 (23)	2 (10)	7 (17)	19 (29)	4 (36)	.12

*ACE* angiotensin-converting enzyme; *ESV* end-systolic volume; *ICD* implantable cardioverter-defibrillator; *LBBB* left bundle branch block; *NYHA* New York Heart Association; *SD* standard deviation; *sec.* secondary

* Results are presented as *n* (%), unless otherwise indicated. Significant results are printed in bold (Bonferroni’s corrected *p* value = 0.05/72 = 6.9e^−4^)

^†^ *p* values were obtained using adjusted Step-3-*Dependent* analysis procedures in Latent GOLD 5.0. All other values were observed values obtained using SPSS

^‡^Defined as primary education versus secondary education and higher

^§^Upgrade from another implantable device, either (biventricular) pacemaker or ICD without cardiac resynchronization therapy

^||^Echocardiographic response was defined as a decrease in left ventricular end-systolic volume of ≥15 % measured at 6-month follow-up

^#^Comorbidity = atrial fibrillation, chronic obstructive pulmonary disease, diabetes, or renal failure

## Discussion

This is the first study to examine the trajectories and associates of disease-specific health status in HF patients receiving a CRT-D device. Latent class analyses identified five trajectories for physical health status and quality of life, and four trajectories for social limitation. All health status trajectories showed an initial improvement in the first 2 months post-implantation, after which most trajectories displayed a stable pattern between short- and long-term follow-up. Low educational level, NYHA class III/IV, smoking, no use of beta-blockers, use of psychotropic medication, anxiety, depression, and Type D personality were found to be associated with poorer health status in univariate/unadjusted analyses. The relationship between ICD shocks/use of amiodarone and health status was less clear. Interestingly, subgroups of patients (12–20 %) who reported poor health status at baseline improved to a good health status level at 2-month follow-up, with these patients being able to retain their improved health status up to 14 months post-implantation.

The results of the present study indicate that levels of disease-specific health status vary considerably across subgroups of CRT-D patients, which is not surprising given the inherent heterogeneity of HF and differences in patients’ response to CRT. However, these subgroups would not have been identified if we had only calculated changes in health status for the total sample.

The finding of early improvement in disease-specific health status after CRT-D implantation followed by stabilization between short- and long-term follow-up is in line with a recent study examining mean and individual health status scores of HF patients receiving a left ventricular assist device [17]. The early health status improvement could be the result of incipient reverse remodeling and enhanced exercise capacity induced by the implanted device. However, it could also represent a study bias, as patients participating in research might exhibit better compliance with respect to intake of medication, recommended health behaviors, etc. Finally, a placebo effect could contribute to the early health status improvement observed after device implantation. Irrespective of the cause(s) of this early improvement, our results indicate that levels of disease-specific health status at short-term follow-up are a good indicator of experienced health status at long-term follow-up. However, this needs to be confirmed in future studies, before any implications for clinical practice can be drawn with respect to advocating a one-time assessment of patient-reported health status rather than multiple assessments.

With respect to demographic characteristics, a lower educational level was found to be a significant associate of poorer health status trajectories. This result is in agreement with the finding of an earlier HF study, suggesting that poorly educated patients [18] may require different or additional interventions to improve their health status. In addition, female gender was identified as a variable distinguishing between patients improving versus not improving from impaired to good health status between baseline and short-term follow-up. Female patients might be more prone to experience placebo effects and therefore have a greater chance of being in the improved health status trajectory compared with men. Moreover, women have shown greater echocardiographic evidence of reverse cardiac remodeling after CRT than men [19].

 With respect to clinical characteristics, only NYHA III/IV classification was found to be clearly related with classification into poorer health status trajectories, which is in line with the findings of previous HF studies in which a higher NYHA classification independently predicted impaired HF-specific health status [20, 21]. Furthermore, results from the HF-ACTION trial found that NYHA class III was associated with a 12.73-point lower KCCQ score than NYHA II [22]. However, NYHA classification has been criticized for its interrater reliability and validity problems, and most of the variation in health status cannot be explained by NYHA class alone [23]. Hence, assessment of HF-specific health status might have additional value in clinical practice to assess patients’ functional status. Although we found a significant association between ICD shocks and classification into health status trajectories, we could not identify a clear direction of the relationship with the highest percentage of shocks found in patients reporting adequate to good health status. This corroborates earlier findings on the influence of ICD shocks on patient-reported outcomes, which are mixed [24]. Finally, with respect to echocardiographic CRT response, our research group has demonstrated a large discrepancy between echocardiographic response and health status improvement after CRT [25]. However, although echocardiographic response was not identified as an associate of poorer health status in the current study, patients reporting the lowest health status levels over time also showed the lowest percentage of response. Furthermore, echocardiographic response was identified as a variable distinguishing between patients improving versus those not improving from poor to good health status between baseline and short-term follow-up. So, patients reporting persistently poor health status after implantation might constitute end-stage HF patients, with enlarged hearts that are ‘beyond repair.’

Classification into poorer disease-specific health status trajectories was found to be particularly associated with patients’ psychological profile (i.e., use of psychotropic medication, anxiety, depression, and Type D personality) and less with their clinical status (except for NYHA classification), which seems to be consistent with earlier research [17, 20, 26, 27]. Identification of patients with a vulnerable psychological profile would provide the opportunity to offer them appropriate treatment. It seems feasible to simultaneously screen for patients’ health status and their personality. For patients reporting poor health status without experiencing anxiety/depression or having a Type D personality, cardiac rehabilitation may suffice. While for patients reporting poor health status and having a distressed personality profile, additional psychological and behavioral intervention may be desirable. Anxiety and depression may be improved by cognitive behavioral therapy, mindfulness, relaxation therapy, and supplementary pharmacotherapy depending on patients’ preferences and needs, while intervention strategies for Type D could focus on improvement in mood, health status, health-related behaviors, and interpersonal functioning [28].

This study is limited by the relatively small patient sample, making it underpowered to perform multivariable analyses to examine which demographic, clinical, and psychological characteristics independently predict health status class membership. However, the present study also has several strengths, including the repeated assessment of disease-specific health status at four time points and the use of a novel and innovative latent class regression technique, which permits the identification of patients reporting persistently low health status, who may need additional care above and beyond standard HF management.

## Conclusion

The results of the present study indicate that levels of disease-specific health status vary considerably across subgroups of CRT-D patients. Classification into poorer disease-specific health status trajectories seems to be particularly associated with patients’ psychological profile and NYHA classification, although this conclusion should be interpreted with caution as no multivariate analyses could be performed. Our results do suggest that individual differences in patients’ profile should be considered in the timely identification of patients who are at increased risk of poor health status. This is of utmost importance, since poor patient-reported health status has been shown to predict mortality and rehospitalization in HF patients independent of traditional risk factors. The timely identification and monitoring of CRT-D patients with poor disease-specific health status (a KCCQ score < 50) and/or a distressed psychological profile (anxiety, depression and/or Type D personality) is paramount, as they may benefit from cardiac rehabilitation in combination with psychological intervention.
